# Enhanced polychronization in a spiking network with metaplasticity

**DOI:** 10.3389/fncom.2015.00009

**Published:** 2015-02-05

**Authors:** Mira Guise, Alistair Knott, Lubica Benuskova

**Affiliations:** Department of Computer Science, University of OtagoDunedin, New Zealand

**Keywords:** metaplasticity, STDP, spiking network, polychronous neural group, memory, spike latency, synaptic weight, synaptic drive

## Abstract

Computational models of metaplasticity have usually focused on the modeling of single synapses (Shouval et al., 2002). In this paper we study the effect of metaplasticity on network behavior. Our guiding assumption is that the primary purpose of metaplasticity is to *regulate synaptic plasticity*, by increasing it when input is low and decreasing it when input is high. For our experiments we adopt a model of metaplasticity that demonstrably has this effect for a single synapse; our primary interest is in how metaplasticity thus defined affects network-level phenomena. We focus on a network-level phenomenon called *polychronicity*, that has a potential role in representation and memory. A network with polychronicity has the ability to produce non-synchronous but precisely timed sequences of neural firing events that can arise from strongly connected groups of neurons called polychronous neural groups (Izhikevich et al., 2004). Polychronous groups (PNGs) develop readily when spiking networks are exposed to repeated spatio-temporal stimuli under the influence of spike-timing-dependent plasticity (STDP), but are sensitive to changes in synaptic weight distribution. We use a technique we have recently developed called Response Fingerprinting to show that PNGs formed in the presence of metaplasticity are significantly larger than those with no metaplasticity. A potential mechanism for this enhancement is proposed that links an inherent property of integrator type neurons called spike latency to an increase in the tolerance of PNG neurons to jitter in their inputs.

## 1. Introduction

The term *metaplasticity* describes the ability of neurons to modulate their overall levels of synaptic plasticity as a function of recent inputs. Models of LTP/LTD induction that include a metaplastic mechanism have been around for some time, with the Bienenstock, Cooper and Munro (BCM) learning rule and its sliding modification threshold being a significant early influence (Bienenstock et al., 1982). The BCM rule provides a rate-dependent model of the tipping point between LTD and LTP induction based on instantaneous neural firing rates. More recently, Izhikevich and Desai (2003) have combined the BCM rule with spike-timing-dependent plasticity (STDP), a learning rule that takes the precise spike timing of pre- and post-synaptic neurons into account. The addition of a BCM sliding modification threshold to an STDP learning rule has also been used to explain experimental data showing hetero-synaptic LTD of untetanized inputs in a model of a hippocampal dentate granule cell (Benuskova and Abraham, 2007). The precise mechanism behind metaplasticity is still an open question despite receiving much recent attention (for a review see Abraham, 2008). Intensive research into the cellular processes behind metaplasticity has uncovered multiple mechanisms that both cooperate and compete, with the balance between the various mechanisms varying between different brain regions.

Models based on BCM define a *modification threshold* for LTP/LTD induction that is dynamically altered as a function of previous post-synaptic spike activity. When spiking activity increases, the modification threshold is also increased and it therefore becomes harder to induce subsequent LTP and easier to induce LTD. A decrease in spiking activity produces the opposite effect, with LTP induction becoming easier and LTD induction becoming more difficult. The BCM learning rule defines a single modification threshold, while later versions have defined separate thresholds for LTP and LTD (Ngezahayo et al., 2000). In one example of a single threshold model (taken from Benuskova and Abraham, 2007), the relationship between the modification threshold and synaptic change is defined as follows:

(1) $$\begin{array}{l} A_{LTP}(t)=A_{LTP}(0)(1/\theta_{M}(t))\\ A_{LTD}(t)=A_{LTD}(0)(\theta_{M}(t)) \end{array}$$

where θ_*M*_(*t*) is the current value of the modification threshold, *A_LTP_*(0) and *A_LTD_*(0) are the baseline amplitudes of synaptic change, and *A_LTP_*(t) and *A_LTD_*(t) are the new amplitudes. Typically, these models assume that the metaplastic modification threshold is determined primarily by the post-synaptic firing rate (e.g., Beňušková et al., 2001), although this assumption is still open to debate (Hulme et al., 2012). Shouval et al. (2002) suggest that the modification threshold is more directly set by the levels of intracellular *Ca*^2+^ while (Izhikevich and Desai, 2003) suggest that synaptic size might also be an influence.

In a synapse whose learning is governed by a spike-timing-dependent plasticity (STDP) rule, the direction and magnitude of neural plasticity is determined not only by factors that govern the level of synaptic input, but also by the precise timing of pre-synaptic and post-synaptic spikes. Changes in synaptic plasticity cannot therefore be predicted from either the post-synaptic firing rate or the total synaptic input alone (Izhikevich and Desai, 2003). In this scenario, the conditions under which the modification threshold should be modified relate to the consistency of these timings in the recent history of the synapse. We use the term *synaptic drive* to describe these conditions: strongly correlated spike trains with pre- before post-synaptic spike timings are defined as producing a positive drive on synaptic plasticity, whilst post- before pre-synaptic spike trains with identical firing rates are defined as having a negative synaptic drive.

In the current paper we define a model of metaplasticity that is determined by the direction and magnitude of synaptic drive, and also by the size of each of the synaptic connections onto the cell (Delorme et al., 2001; Guetig et al., 2003). The model is therefore both spike-timing-dependent and reactive to synaptic weight extremes i.e., it resists synaptic pruning and opposes synaptic weights that grow too large. We have chosen to define the metaplastic modification threshold in this model as a cell-level property that integrates the changes in plasticity that are occurring at each synapse. The choice of a cell-level property, rather than defining a modification threshold at each synapse, allows the metaplasticity model to be integrated into a larger model of network behavior and is supported by a recent finding that metaplastic effects can be seen in non-primed dendritic compartments (Hulme et al., 2012). Previous computational models of metaplasticity have typically focused on the modeling of single synapses, although reports on the effect of metaplasticity at network-level have recently started to appear (Clopath et al., 2010; Zenke et al., 2013). Like any computational model of the synapse, the model of metaplasticity we use in our experiments is motivated by a mixture of mechanistic and computational considerations. Some components in the model aim to account for specific empirically identified biological mechanisms in the synapse. Other components are included to implement a particular theoretical claim about the *function* of metaplasticity—namely that it serves to regulate synaptic plasticity, by increasing it when input is low, and decreasing it when it is high (see e.g., Hulme et al., 2012; Zenke et al., 2013). In both cases, the model we use is heavily based on existing models of synaptic plasticity, though it also includes novel mechanistic and novel functional components.

The primary focus of our study is the impact of metaplasticity on an empirically observed property of spiking neural networks called *polychronicity*. Polychronous neural groups (or PNGs) are connected groups of neurons that can be activated together to produce polychronization, a non-synchronous but precisely timed sequence of neural firing events (Izhikevich et al., 2004). These stimulus-specific firing signatures form reproducible patterns that are observable in the firing data generated by the network. Polychronization requires that the connection weights between PNG neurons be *adapted* to support a sequential chain of neural firing (Martinez and Paugam-Moisy, 2009). With an STDP learning rule, this adaptation occurs readily when spiking neural networks are exposed to repeated spatio-temporal stimuli. The STDP rule combined with repeated stimulation potentiates intra-group connection weights and prunes non-contributing connections, leading to the preferential selection of stimulus-dependent polychronous groups (Izhikevich et al., 2004).

Given that polychronous groups evolve via selective enhancement of the connections between PNG neurons, it is often assumed that the stability of adapted PNGs over an extended period requires that these same connections be maintained. However, polychronicity requires only that the combined input to PNG neurons be sufficient to produce firing within a precise temporal window. Theoretically therefore, PNG neurons can remain stable within the group even if the weight value on some of their afferent connections wanders randomly, so long as other input connections evolve their weights to compensate. This proposed independence of PNG stability from the weight values of specific synapses leaves the weights free to support other aspects of the network dynamics such as competing or co-activating PNGs, the maintenance of the balance between excitation and inhibition (van Vreeswijk and Sompolinsky, 1996; Vogels et al., 2005), and *mixture states* of synchronization and desynchronization in the network firing activity (Lubenov and Siapas, 2008).

Evidence for polychronicity in biological networks has been technically difficult to establish, although precise spatio-temporal firing patterns observed in rat and monkey cortical neurons provide some supporting evidence (Villa et al., 1999; Shmiel et al., 2006). However, in simulated networks the process of isolating structural PNGS or detecting PNG activation is straightforward (e.g., Izhikevich et al., 2004; Martinez and Paugam-Moisy, 2009). We use a recently developed technique called Response Fingerprinting to test whether polychronicity both persists and is stable within our model metaplastic regime (Guise et al., 2014).

A modified STDP rule that includes a metaplastic mechanism is likely to have a significant effect on PNG formation and may also be more biologically plausible than existing STDP rules. Lazar et al. (2007) report improvements in both network performance and stability using a combination of intrinsic plasticity with STDP to produce a reduction in synaptic saturation. A metaplastic modification to the STDP rule has the potential to maintain synaptic weights more centrally in the range and may therefore produce a similar performance advantage. However, the formation of polychronous groups has a significant effect on synaptic weights, resulting in a characteristic bimodal weight distribution that opposes this predicted centralizing effect. Polychronizing pathways are very dependent on strong connections that support convergent input to PNG neurons, and therefore any network mechanism that affects the synaptic weight distribution is predicted to have a significant effect on PNG formation. Given the opposing effects of PNG formation and metaplasticity on synaptic weight distributions, it is not clear whether PNG formation will be supported in networks with the new metaplastic mechanism, and if it is supported, what the effect will be on PNG size.

## 2. Methods

### 2.1. Metaplasticity model

#### 2.1.1. Methodological preliminaries

As mentioned in the introduction, the model of metaplasticity we implemented in our experiments was designed to accommodate a mixture of mechanistic and computational considerations. The computational considerations are uppermost: we assume that the key purpose of the metaplastic mechanism we are modeling is to *regulate synaptic plasticity*, by limiting the range of weights within any given synapse, forcing weights away from both their upper and lower extremes. Accordingly, a key design goal for our computational model is that it produces this effect. At the same time, we want the model to make as much reference as possible to empirically identified mechanisms in the synapse, so that the regulatory effect can be linked as much as possible to physiological processes. On both counts our model draws heavily on existing models of metaplasticity. A useful point of reference is the model of metaplasticity of Zenke et al. (2013). Like our model, this model implements an assumption that the main purpose of metaplasticity is to regulate synaptic plasticity. However, our model incorporates a slightly different set of mechanistic components to achieve this effect. We will draw attention to these differences as the model is introduced.

One difference to mention straight away is that our model defines a metaplastic modification threshold that is a neuron-level property: the theshold value for a given neuron is computed from a weighted average of the threshold values of its afferent synapses. The model is therefore best described in two sections: a synapse-level model that weights the size of each synapse according to the current direction and magnitude of synaptic change (synaptic drive); and a neuron-level model that is computed as a weighted sum of the individual synaptic values.

#### 2.1.2. Synapse-level model

The metaplasticity model at the level of each individual synapse is defined by a weighting function that computes a weighted value for each synapse. The weighting function takes values representing the current synaptic weight and synaptic drive as arguments, and returns a weighted value representing the resistance to synaptic weight change. The synaptic drive is dependent on both the level of synaptic input and the precise timing of that input relative to a back-propagating dentritic signal. In our simulated network, we approximate the synaptic drive with a synaptic derivative, an instantaneous measure of the direction and magnitude of change at each synapse that is an explicit value from the original network simulation code (Izhikevich, 2006b).

The desired weighting function needs to exert little influence when synaptic weights are within bounds, but must step in with increasing resistance as the weights approach the upper or lower weight limits. One possibility, that we use throughout this paper, is as follows:

(2) $$f(d_{i},w_{i})=r{\text{e}}^{p(map(d_{i}))(w_{i}-min)}-r{\text{e}}^{p(10-map(d_{i}))(max-w_{i})}$$

where:

$$\begin{array}{l} \;p=\text{precision}\\ \;r=\text{resistance}\\ \;min=\text{minimum soft limit}\\ \;max=\text{maximum soft limit }\\ \;d_{i}=\text{derivative of synapse }i,\;d_{i}={\text{ lim}}_{\Delta t\to 0}\frac{\Delta w_{i}}{\Delta t}\\ \;w_{i}=\text{weight of synapse }i\\ map(x)=\{\begin{array}{ll} 0 & \text{if }0.5(x+10)<0\\ 10 & \text{if }0.5(x+10)>10\\ 0.5(x+10) & \text{if }0\leq 0.5(x+10)\leq 10 \end{array} \end{array}$$

**Figure 1:**
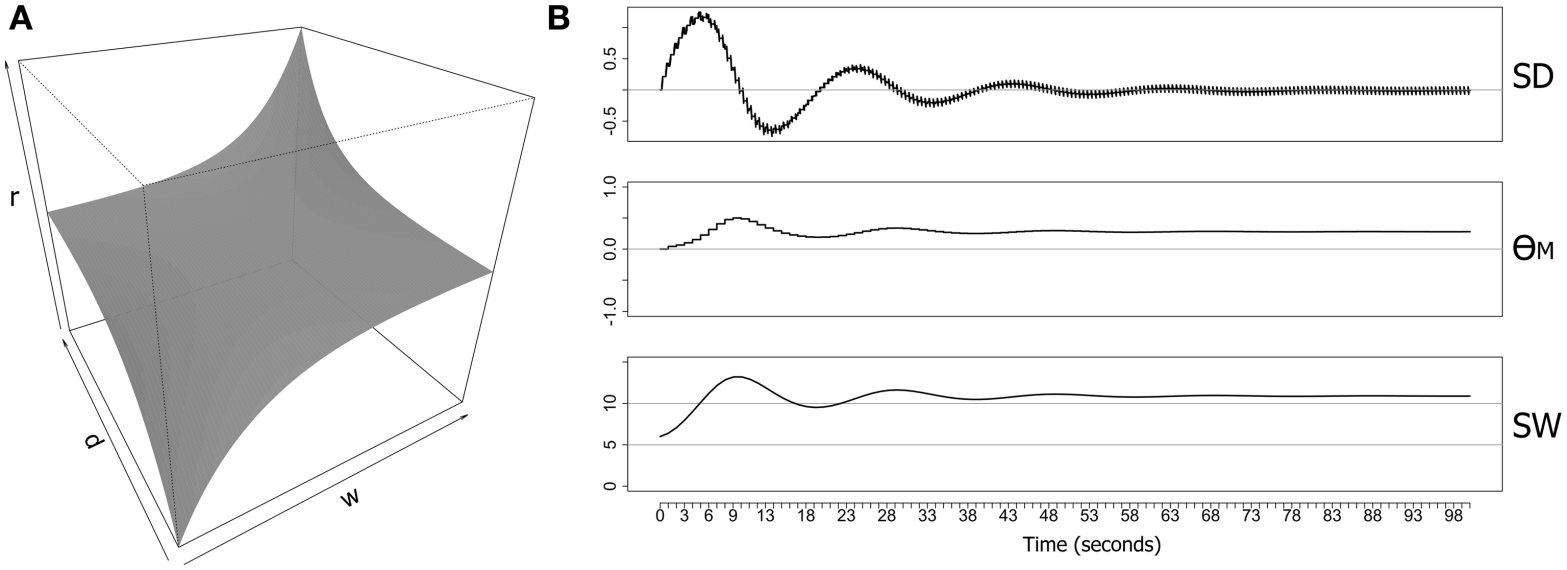
**Metaplasticity model for a single synapse showing the weighting function and an example of some computed output values**. **(A)** A symmetrical weighting function based on Equation (2) with parameters (*r* = 0.1, *p* = 0.05) **(B)** Data generated over 100 s from an implementation of the neuron-level metaplasticity model with a *single synapse*. The model consisted of two excitatory RS-type Izhikevich neurons (Izhikevich, 2006a) with a single synaptic connection (delay = 1 ms; initial weight = 6.0 mV; max. synaptic weight = 15.0 mV). Both neurons were directly stimulated with two evenly-spaced bursts of six pulses at 5 Hz delivered every second, with each pre-synaptic pulse leading the post-synaptic pulse by 5 ms (producing positive synaptic drive). Weighting function parameter values (Equation 2) were (*r* = 0.1, *p* = 0.05). SD, synaptic derivative; θ_*M*_(*t*), metaplastic modification threshold; SW, synaptic weight (mV).

The *map* function maps the normal range of synaptic derivative values (−10 to +10) into the range 0–10, and clips values outside of this normal range. The precision (*p*) and resistance (*r*) parameters control the curvature and amplitude of the function. The weight limits are determined by the parameters *min* and *max* that specify uncapped *soft* limits rather than capped hard limits on synaptic weights. Figure 1A shows the full picture for both parameters (*d_i_* and *w_i_*) over the weight range 0–10 and limiting the range of the synaptic derivative to ±10. Most combinations of *d_i_* and *w_i_* generate a weighting term that is close to zero, and the resulting surface is therefore largely flat for these combinations. However, the surface exponentially rises and falls in opposing corners, producing maximum resistance to increases in synaptic weight when the synaptic drive is positive and the weight is already large, and maximum resistance to decreases in synaptic weight when the synaptic drive is negative and the weight is already small. However, the function generates little resistance to large weights if the synaptic drive is negative, or to small weights if the synaptic drive is positive, providing no impediment to migration of synaptic weights away from the weight limits.

#### 2.1.3. Neuron-level model

The *modification threshold (θ_*M*_)* in this model is a neuron-level property that determines the ease of subsequent synaptic change. Unlike previous models, the modification threshold is dependent on synaptic drive and therefore only indirectly dependent on the post-synaptic spike rate or the total input. When synaptic drive is strongly positive, θ_*M*_ increases, and subsequent LTP induction becomes harder (and LTD induction becomes easier). A negative synaptic drive produces the opposite effect by causing θ_*M*_(*t*) to decrease.

The modification threshold is computed as a range-limited average of the weighting function output for each of the afferent synaptic connections. The weighting function outputs represent a *weighted synaptic derivative* for each synapse, and the average therefore represents the integrated synaptic drive across all synaptic inputs. The weighting mechanism assumes that a global metaplastic signal interacts with the local conditions (particularly the synaptic size) at each synapse.

Given a weighting function *f*(*d_i_, w_i_*), the modification threshold is computed as follows:

(3) $$\theta_{M}(t)=\tanh\;\left(I\frac{\sum_{i=1}^{n}f(d_{i},\;w_{i})}{n}\right)$$

where:

$$\begin{array}{l} \;I=\text{inertia}\\ \;n=\text{number of synapses}\\ f(d_{i},w_{i})=\text{a weighting function} \end{array}$$

The hyperbolic tangent limits the range of the modification threshold to ±1, while the *inertia* parameter controls the rate of change of θ_*M*_ i.e., the sensitivity of the modification threshold to small changes in the average weighted synaptic derivative. Here we are assuming a biophysical process that maps a wide input range into a narrower response range such as in the proposed power-law relationships between stimulus strength and perceived intensity (MacKay, 1963).

Equations (2) and (3) are novel elements in a model of metaplasticity, in that they assume a generalized postsynaptic activity function rather than the ‘spike counter’ assumed by existing models (see e.g., Benuskova and Abraham, 2007; Zenke et al., 2013). However, this departure is justified in the light of recent evidence that synaptic plasticity can be homeostatically regulated by the cell-wide history of synaptic activity through a calcium-dependent but action potential-independent mechanism (Hulme et al., 2012).

Given the modification threshold, the amplitudes of synaptic change in LTP and LTD can now be calculated as follows:

(4) $$A_{LTP}(t)=A_{LTP}(0)-(A_{LTP}(0)\theta_{M}(t))$$

(5) $$A_{LTD}(t)=A_{LTD}(0)+(A_{LTD}(0)\theta_{M}(t))$$

The two equations in (4) are symmetrical by design: if θ_*M*_(*t*) is positive then the LTP amplitude (*A_LTP_*) decreases, and the LTD amplitude (*A_LTD_*) increases by the same proportion. Unlike the equation described by (Benuskova and Abraham, 2007) (see Equation 1), the LTP and LTD amplitudes in Equation (4) are modified in direct proportion to the modification threshold (θ_*M*_) and the current baseline amplitudes. In contrast, each of the amplitudes of synaptic change in the equation of Benuskova and Abraham (2007) differ in their relationship to the modification threshold: *A_LTP_* is inversely proportional to θ_*M*_, while *A_LTD_* is directly proportional. If spike activity is low but consistent, the equation of Benuskova and Abraham (2007) has the potential to create a dramatic imbalance between *A_LTP_* and *A_LTD_* that allows synaptic weight to increase without limit. Clopath et al. (2010) and Zenke et al. (2013) introduced models in which only the LTD amplitude is metaplastically modified and the LTP amplitude stays constant. However, we do not have any neurobiological evidence why only LTD would be subject to homeostatic control and LTP not, therefore we assume that magnitudes of both are metaplastically modified.

Metaplasticity models based on post-synaptic spike rate restrict the modification threshold to positive values. However, in the current model (based on synaptic drive) and in models based on post-synaptic membrane potential, both positive and negative values are allowed (Ngezahayo et al., 2000). We limit the range of the modification threshold to ±1 (above) so that both the LTP and LTD amplitudes have the range 0–2 times the baseline amplitudes, and have default values of *A_LTP_*(0) and *A_LTD_*(0) respectively. Although there is no explicit limit on synaptic growth in Equation (4), the symmetry between the equations for LTP and LTD limits the degree of imbalance between the two.

It is worth emphasizing that because the metaplastic modification threshold is calculated from an average of the values returned by the weighting function, the resistance to synaptic weights that near the limits also applies *only on average*. Therefore, individual synapses are allowed to grow without limit so long as the average across all synaptic inputs is within the allowed weight range. Synaptic pruning can still therefore occur, even if the value for weight limit resistance in Equation (2) is large. Likewise, while individual synapses are allowed to grow large, they will increasingly dominate the weighted average as they grow, providing an implicit limit to their growth.

### 2.2. Network simulations

#### 2.2.1. Networks

For network simulations we use a spiking neural network platform we have developed called Spinula (Guise et al., 2013) that is based on the reference implementation from Izhikevich (2006b). Twenty different networks were generated for these experiments, with each network composed of 1000 Izhikevich neurons (800 excitatory RS type and 200 inhibitory FS type). Within each network each simulated neuron was connected to 100 randomly selected post-synaptic neurons with the restriction that inhibitory neurons were connected to excitatory neurons only. Inhibitory connections were assigned a 1 ms conduction delay while excitatory connections were randomly assigned a delay in the range 1–20 ms. Connection weights were initialized to the values +3.0 mV (for excitatory weights) and −2.0 mV (for inhibitory weights). Each network was then matured for 2 h by exposure to a 1 Hz random input under the influence of a spike-timing-dependent plasticity (STDP) rule. The STDP rule was temporally asymmetric and with parameters as in (Izhikevich, 2006a) i.e., *A*_+_ = 0.1 and *A*_−_ = 0.12. Random input was generated by an independent Poisson process on each neuron.

#### 2.2.2. Training

**Figure 2:**
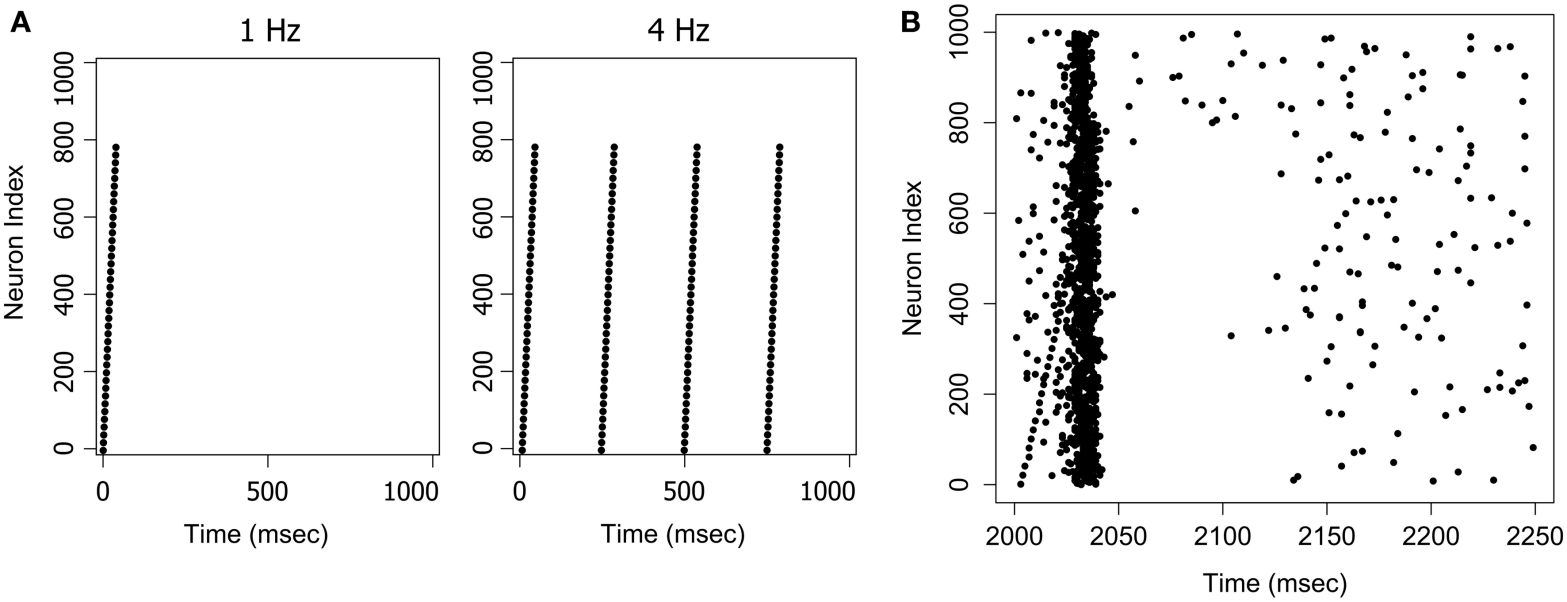
**Examples of stimuli and the stimulus response**. **(A)** The *Ascending* pattern as a 1 Hz or 4 Hz stimulus. **(B)** The response to a 4 Hz stimulus. A network trained on the *Ascending* pattern was repeatedly presented with the same pattern at 4 Hz with a 1 Hz random background. The figure shows a randomly selected response frame between *t* = 2000 and *t* = 2250 ms. The input pattern can also be seen as an ascending sequence of firing events in the first 40 ms of the frame. The network responds to this pattern with an avalanche-like burst of activity, that builds as the signal arrives, and then terminates quite suddenly when activity in the pool of inhibitory neurons reaches a critical threshold.

Networks were trained on a 5 Hz stimulus with a 1 Hz random input for 180 s (internal simulation time). Guise et al. (2014) have previously reported that PNG size reaches a plateau within around 2 min with this training protocol. Each stimulus was composed of forty firing events arranged in an ascending pattern (see Figure 2 for an example of the *Ascending* pattern, and Guise et al., 2014, for further details). Metaplasticity-related parameters were *r* = 0.1; *p* = 0.5; inertia = 0.2; maximum synaptic weight = 10.0 mV (hard limit). Following training, synaptic weight distributions were generated from the saved synaptic weights for each network. The number of neurons participating in PNG activation was assessed by generating a Response Fingerprint for each network.

#### 2.2.3. Response fingerprinting

The effect of the metaplasticity model on large networks of 100,000 synapses was examined using a technique we have recently developed called *Response Fingerprinting* (Guise et al., 2014, for implementation details see Guise et al., 2013a). A Response Fingerprint is a probabilistic representation of PNG activation that describes the spatio-temporal pattern of firing within a network in response to an input stimulus. It consists of a set of time windows within which specified neurons are likely to fire with empirically determined probabilities; information can be combined across time windows using Bayesian techniques to derive an aggregate estimate of the likelihood of the stimulus. The effect of metaplasticity on the ability of a network to polychronise was assessed by comparing the Response Fingerprints generated by the network with and without metaplasticity enabled. Response Fingerprints were generated by profiling the firing event data in the presence of a 1 Hz random background and identifying peaks in the histograms using a final consistency threshold of 0.75, a measure of the consistency of spiking within each peak region.

#### 2.2.4. Connection activation

The presentation at fixed intervals of a known stimulus (one on which a network has been trained) produces a regular pattern of firing reflecting the activation of a PNG. The network connections can be partitioned into those that are regularly activated by the stimulus and those that are not, allowing an examination of the differential effect of metaplasticity on connections that participate in PNG activation vs. non-participating connections. The partitioning procedure involves attempting a *fit* for each of the 100,000 connections in each network to firing data generated from the network in response to the stimulus: for each connection and each pair of firing events in the firing data, we label the connection as *active* if *connection length* ≤ *time difference* ≤ *connection length*+*jitter*, otherwise the connection is labeled *non-active* i.e., if the time difference between firing events is longer than the connection length by *some small amount* then the pre-synaptic spike was probably a contributor to the post-synaptic firing event and can be considered to be a part of the PNG activation. The allowed variation or *jitter* is typically set to 2 ms.

#### 2.2.5. Input space response

The Input Space Response (ISR) of a neuron is produced by varying the firing times of each of the afferent neurons over a defined range and recording which of the resulting spatio-temporal patterns produces consistent firing of the post-synaptic neuron. This *input space* of potential firing patterns has the same dimension as the number of inputs to the target neuron, and the *active input space* is the subset of the input space that produces firing of the target. For example, neuron 4 in **Figure 8** has three input neurons. If the firing times of 1, 2 and 3 are systematically varied over the range 1–20 ms (keeping connection delays fixed) then the combination of all inputs produces a 20 × 20 × 20 cube of spatio-temporal patterns, only some of which produce firing of neuron 4. With just three inputs the cube may conveniently be flattened to two dimensions by taking the difference between each pair of firing times i.e., (*t*1 − *t*2) and (*t*2 − *t*3), where (*t*1, *t*2, *t*3) are the firing times of neurons 1, 2, and 3. Only two difference pairs are required as the remaining difference (*t*1, *t*3) is constrained by the other two. This 2D projection has the additional benefit of removing redundancies, as many of the patterns in the original cube are just shifted versions of the same spatio-temporal pattern.

## 3. Results

The intention of the metaplasticity model was to force the synaptic weight values away from the extremes and toward the middle of the weight range. However, in networks with many afferent connections we might expect this effect to be diluted by the large number of synaptic inputs onto each neuron. Nevertheless, the metaplasticity model attempts to maintain a central weight for each synapse *on average*, and might therefore be expected to increase the number of non-saturated weights in the network. Significantly, this predicted effect opposes the bimodal weight distributions observed during PNG formation. It is unclear which of these effects will be stronger, the PNG-formation effect that moves synaptic weights toward the limits through STDP, or the metaplasticity effect that moves weights toward the center of the range.

### 3.1. Overall effects

#### 3.1.1. Weight distributions

**Figure 3:**
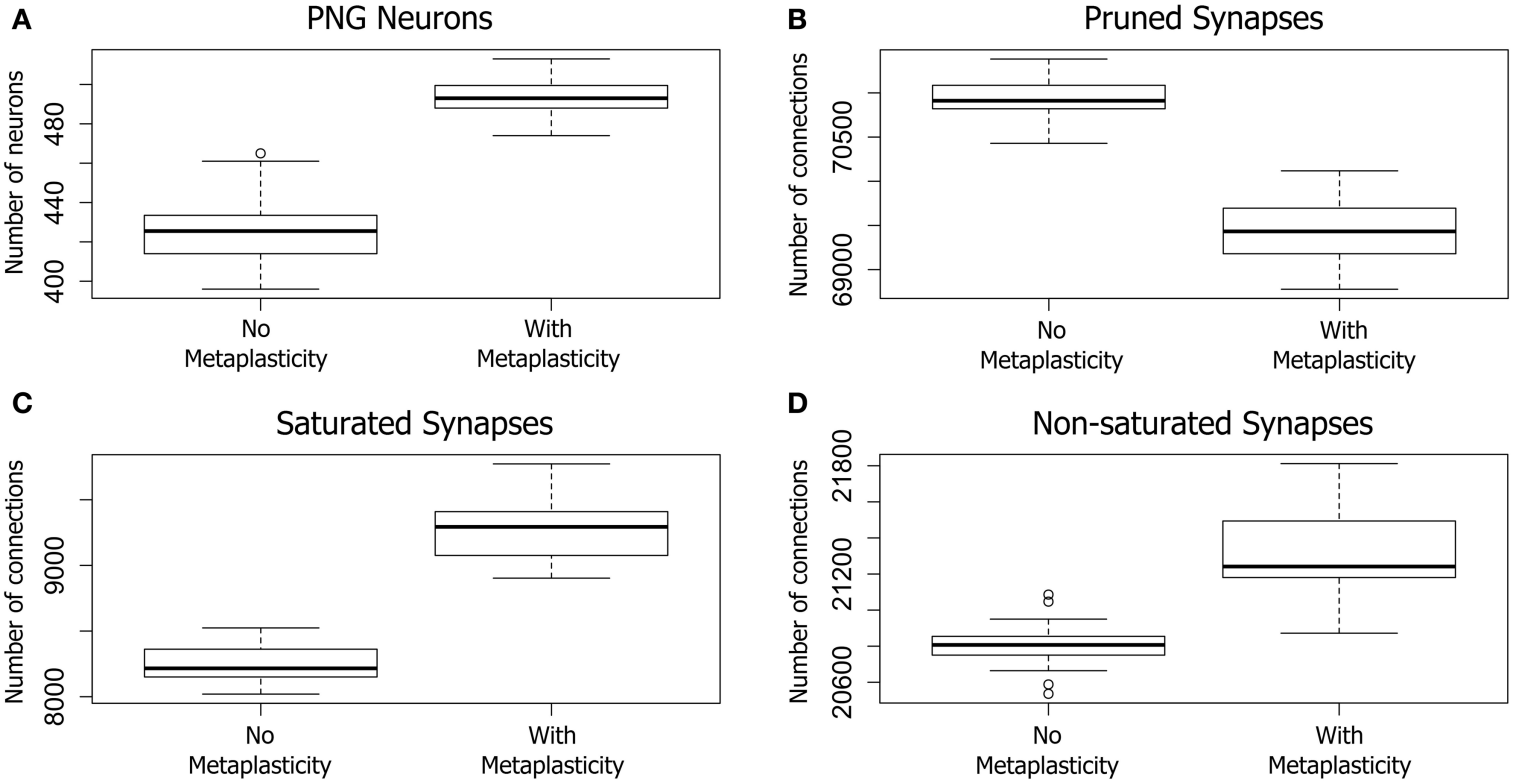
**The effect of metaplasticity on twenty large networks showing the change in PNG size (A), or changes in synaptic weight distributions (B–D) with metaplasticity either enabled or disabled**. The boxes in each box-and-whisker plot show the location of the middle 50% of the data, while whiskers show either the maximum (minimum) value or 1.5 times the interquartile range (IQR). Outliers that are outside 1.5 times the IQR are shown as circles (Crawley, 2012). A. Change in the average number of PNG neurons (PNG size). **(B–D)** Change in average synaptic weight distributions. Each of the 80,000 excitatory connections in each network was assigned to one of the following categories: Pruned (synaptic weight of zero), Saturated (maximum synaptic weight), Other (non-zero and non-saturated synaptic weight). Data: PNG Size = (means: with metaplasticity = 493, no metaplasticity = 426) (paired *t*-test: *t* = 15.0106, *p* < 0.001 (2-tailed), d.f. = 19). Pruned = (means: with metaplasticity ≈ 69400, no metaplasticity ≈ 71000) (paired *t*-test: *t* = 18.0874, *p* < 0.001 (2-tailed), d.f. = 19). Saturated = (means: with metaplasticity ≈ 9300, no metaplasticity ≈ 8300) (paired *t*-test: *t* = 20.4666, *p* < 0.001 (2-tailed), d.f. = 19). Non-saturated = (means: with metaplasticity ≈ 21300, no metaplasticity ≈ 20800) (paired *t*-test: *t* = 8.2596, *p* < 0.001 (2-tailed), d.f. = 19).

The results on twenty large networks of 100,000 synapses each are shown in Figure 3. Metaplasticity was found to have a significant effect on the distribution of excitatory synaptic weights in each network (inhibitory weights are non-plastic and are therefore not shown in Figure 3). For convenience, each of the eighty thousand excitatory connections was categorized into just one of three weight groups as follows: synaptic connections with zero weight (*pruned synapses*); connections with the maximum synaptic weight (*saturated synapses*); and the remaining connections that were neither pruned or saturated (*non-saturated connections*). The overall effect of metaplasticity on these networks was a shift in the weight distribution toward larger weights when metaplasticity is enabled.

Pruned synapses were particularly affected (see Figure 3B). The number of pruned synapses dropped significantly when metaplasticity was enabled relative to the number with metaplasticity disabled, producing an increase in the number of *effective connections* (i.e., those with non-zero weight). On average, around 1500 additional connections were added to the network when metaplasticity was enabled and these were distributed between both saturated and non-saturated connections, affecting the counts for these weight groups. The number of saturated connections was therefore significantly increased with approximately 1000 additional connections becoming saturated when metaplasticity was enabled (see Figure 3C). There was also a significant increase in the number of non-saturated connections (Figure 3D): around 500 additional non-saturated connections were observed with metaplasticity enabled, relative to a network with no metaplasticity as originally predicted from the single synapse model.

#### 3.1.2. PNG Size

Of particular relevance to the focus of this paper, metaplasticity also produced a significant increase in the *average PNG size* across networks (see Figure 3A): the number of PNG neurons was significantly higher with metaplasticity enabled than with metaplasticity disabled. There was also a significant increase in the excitatory firing rate measured at the end of the training period when networks were trained with metaplasticity enabled [*t* = 17.7123, *p* < 0.001 (2-tailed), d.f. = 19; mean (enabled) = 6.0; mean (disabled) = 5.1] (results not shown).

### 3.2. Effects on PNG connections

Given the observed increase in PNG size when metaplasticity is enabled, it is worth considering the differential effect of metaplasticity on the weight distributions of connections that do or do not participate in PNG activation. This entails detecting those connections that are regularly activated by the stimulus, allowing the network connections to be partitioned into *PNG connections* (i.e., those that participate in PNG activation) and *non-PNG connections* (i.e., those that do not).

#### 3.2.1. Weight distributions

**Figure 4:**
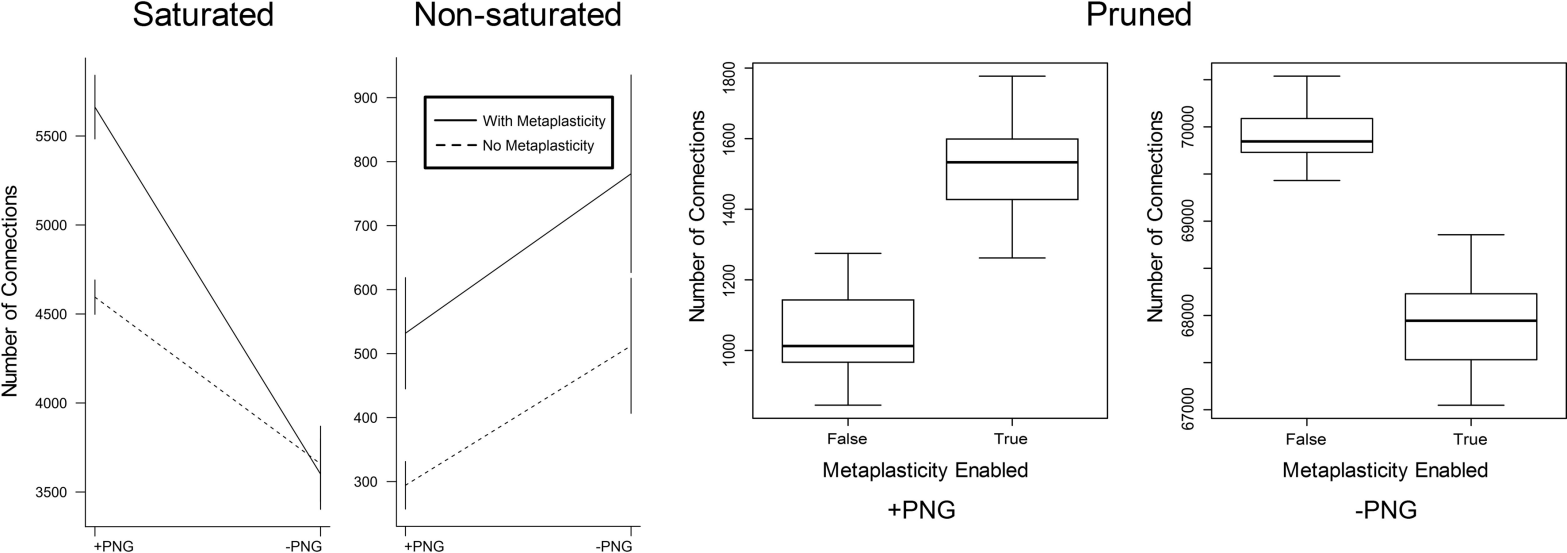
**The effect of metaplasticity on the proportion of PNG vs. non-PNG connections**. Connections were assigned to three categories as in Figure 3. In each weight category, connection numbers were counted for each combination of PNG participation and metaplasticity status i.e., *PNG/with metaplasticity, PNG/no metaplasticity, non-PNG/with metaplasticity* and *non-PNG/no metaplasticity*. Each of the four plotted values in each graph represents the mean over twenty different networks with metaplasticity enabled or twenty networks with metaplasticity disabled. The vertical bars on each plotted value represent one standard deviation above and below each plotted mean. However, for the Pruned data the activated vs. non-activated values are too far apart to be seen clearly using this plotting method. The Pruned data is therefore plotted as two boxplot graphs representing the activated vs. non-activated values, with each boxplot representing the mean and range for the same twenty networks with metaplasticity either enabled or disabled. The interaction between PNG participation and metaplasticity status is significant for the Saturated and Pruned groups but not for the Non-saturated group. Data: Saturated = (means: *PNG/no metaplasticity* 4595; *PNG/with metaplasticity* 5662; *non-PNG/no metaplasticity* 3658; *non-PNG/with metaplasticity* 3601). Non-Saturated = (means: *PNG/no metaplasticity* 294; *PNG/with metaplasticity* 532; *non-PNG/no metaplasticity* 512; *non-PNG/with metaplasticity* 781). Pruned = (means: *PNG/no metaplasticity* 1038; *PNG/with metaplasticity* 1533; *non-PNG/no metaplasticity* 69903; *non-PNG/with metaplasticity* 67891).

The effect of metaplasticity on the proportion of PNG vs. non-PNG connections in each of the weight groups of Figure 3 can be seen in Figure 4. There is a significant interaction between the metaplasticity status of the networks and the PNG participation of the connections for some but not all of these weight groups. Saturated weights increase for PNG connections when metaplasticity is enabled, but not for non-PNG connections. For the non-saturated weight group both PNG and non-PNG connections increase in numbers when metaplasticity is enabled, but with no significant interaction for this weight group. Particularly notable is that, despite the overall decrease in pruned weights observable in Figure 4, the number of pruned weights in the *PNG* group actually increases when metaplasticity is enabled. These effects of metaplasticity are small, but given the strongly recurrent structure of these networks, they might still have important consequences on the network dynamics.

#### 3.2.2. PNG Size

**Figure 5:**
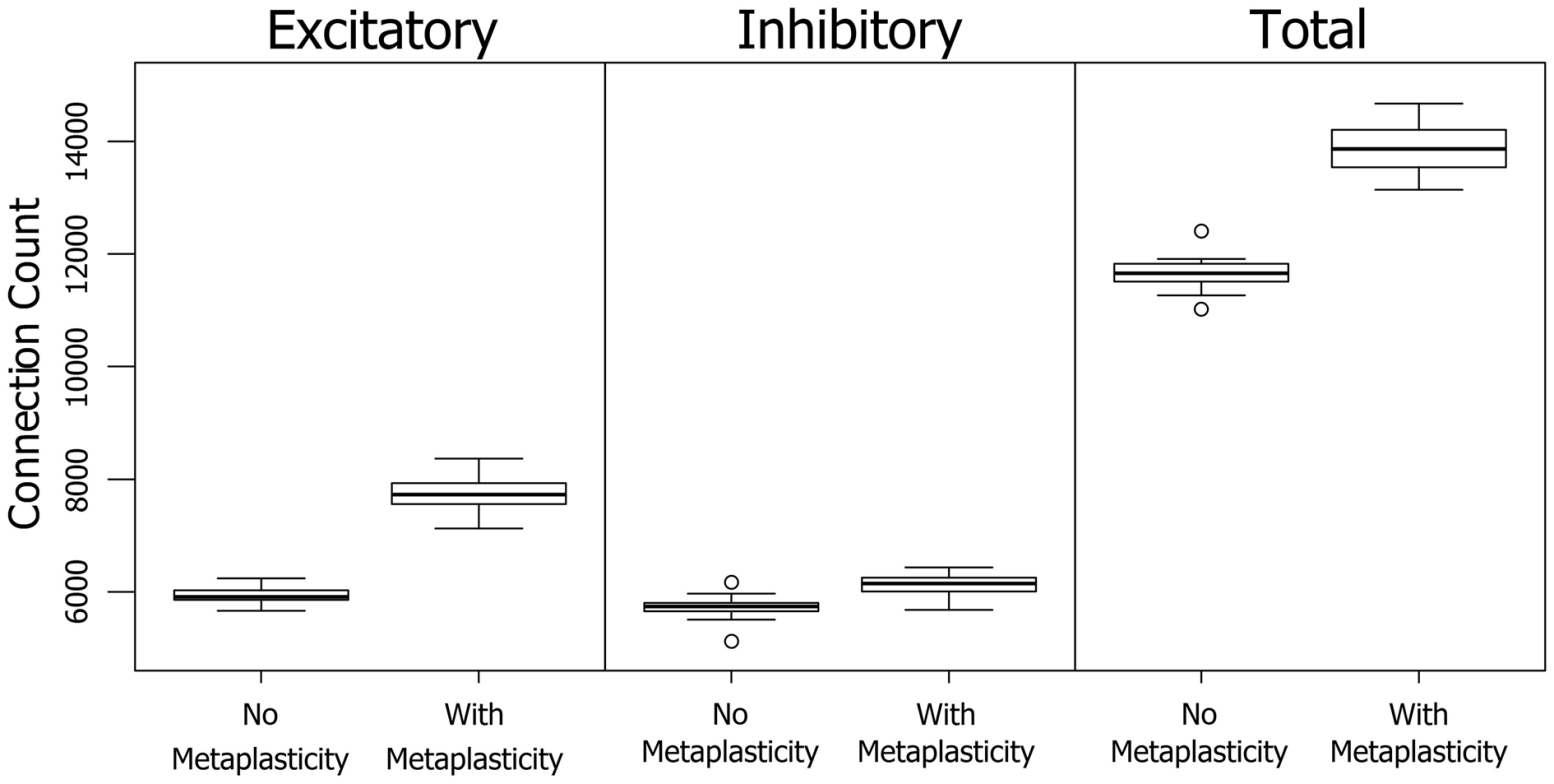
**The effect of metaplasticity on the the number of PNG connections**. Left: Connection counts for *excitatory PNG* connections. Middle: Connection counts for *inhibitory PNG* connections. Right: Total connection counts. Data: Excitatory = (means: +meta ≈ 7730 −meta ≈ 5930) (paired *t*-test: *t* = 27.5989, *p* < 0.001 (2-tailed), d.f. = 19) Inhibitory = (means: +meta ≈ 6120 −meta ≈ 5730) (paired *t*-test: *t* = 7.2883, *p* < 0.001 (2-tailed), d.f. = 19) Total = (means: +meta ≈ 13900 −meta ≈ 11700) (paired *t*-test: *t* = 23.1757, *p* < 0.001 (2-tailed), d.f. = 19).

The results in Section 3.1.2 show a significant increase in the number of neurons involved in PNG activation when metaplasticity is enabled. A technique for partitioning connections allows an alternative view of PNG activation size in terms of the number of participating connections. Figure 5 shows the effect of metaplasticity on PNG connection counts for each of the 20 independent networks in Figure 3. Unsurprisingly, given the previously observed increase in the number of PNG neurons, enabling metaplasticity produces a significant increase in the total number of PNG connections in each network. Interestingly, most of this increase comes from additional excitatory connections that are recruited into the PNG activation when metaplasticity is enabled.

### 3.3. Effects of variation in the metaplasticity parameters

**Figure 6:**
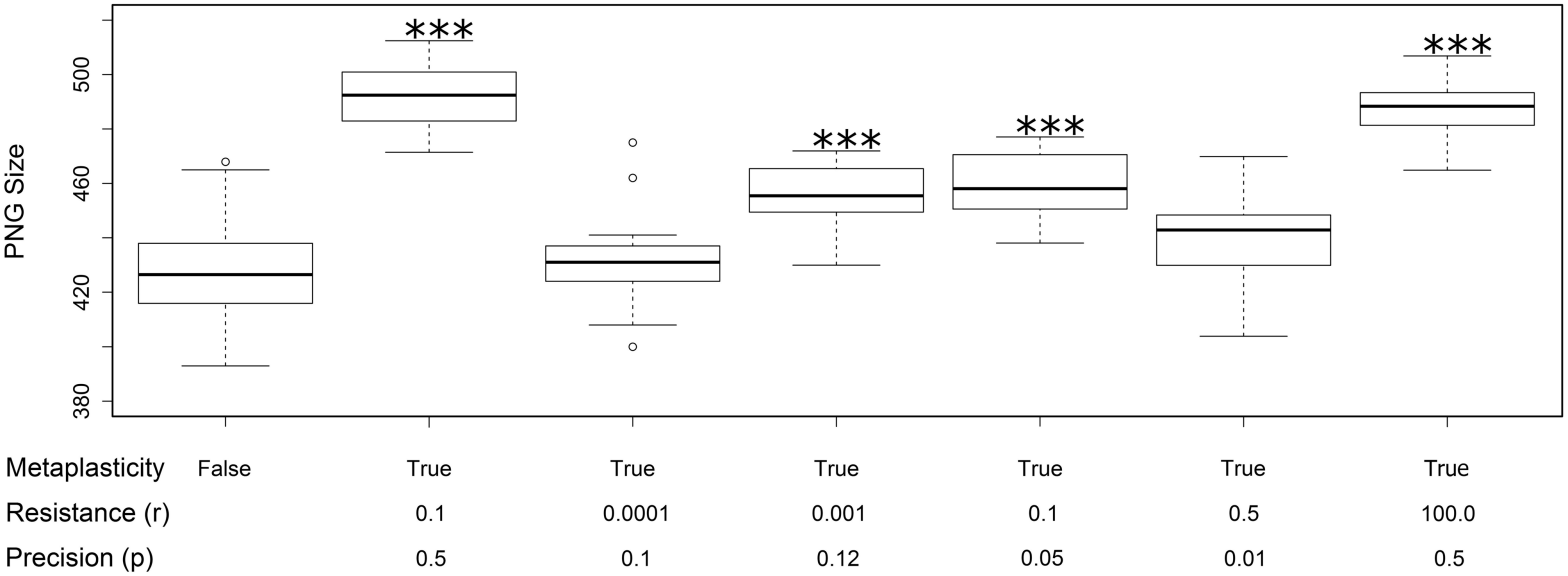
**Changes in the distribution of PNG sizes produced using different values for the metaplasticity parameters *r* and *p***. Each boxplot shows the mean and distribution of PNG sizes produced from twenty different networks using the specified values for resistance and precision. The two left-most plots are taken from Figure 3A: the first shows the PNG sizes produced with metaplasticity disabled and the second shows the PNG sizes produced with the original metaplasticity parameters (*r* = 0.1; *p* = 0.5). The remaining five boxplots show the effect of other parameter values on the PNG size distribution. The significance of these effects are as follows: *r* = 0.0001 and *p* = 0.1: no significance; *r* = 0.001 and *p* = 0.12 (mean = 456: paired *t*-test: *t* = 7.319, *p* < 0.001 (2-tailed), d.f. = 19); *r* = 0.1 and *p* = 0.05 (mean = 459: paired *t*-test: *t* = 7.0517, *p* < 0.001 (2-tailed), d.f. = 19); *r* = 0.5 and *p* = 0.01: no significance; *r* = 100.0 and *p* = 0.5 (mean = 488: paired *t*-test: *t* = 11.2943, *p* < 0.001 (2-tailed), d.f. = 19). The value of the inertia parameter was 0.2 in all cases. ^***^p < 0.001.

All of the effects reported above used the same values for the resistance (*r*) and precision (*p*) metaplasticity parameters (*r* = 0.1 and *p* = 0.5). In this section we briefly discuss some experiments with alternative parameter values. Figure 6 shows the effect of a random selection of alternative values on the PNG size distributions. The size distribution with metaplasticity disabled is shown on the left for comparison. These results allow a few preliminary observations. Firstly, there is a strong interaction between the two metaplasticity parameters: for instance setting *r* to very small values has the same effect as disabling metaplasticity, regardless of the value of *p*. Secondly, metaplasticity certainly has a positive effect on PNG size over a considerable range of values of *r* and *p*, so the effects we observed are not due to fortuitous or carefully tweaked settings of these parameter values.

### 3.4. A role for spike latency?

**Figure 7:**
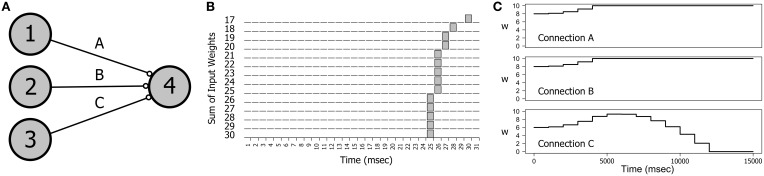
**The effect of spike latency**. **(A)** The network topology: three neurons providing input to a single output neuron. **(B)** Near threshold inputs produce a delayed spiking response. The firing time of neuron 4 decreases as the combined synaptic weights of each of the three inputs is increased. Weights on connections A, B, and C were incremented together in steps of 1 mV in the range 17–30 mV. Connection delays were randomly chosen for each experiment. **(C)** Spike latency explains the switch in synaptic drive that is observed with some combinations of delays on connections A, B, and C. Here a switch in synaptic drive on connection C during training produces an initial increase in synaptic weight followed by a decrease. The training stimulus involved repeatedly firing neurons 1, 2, and 3 together at 5 Hz. Connection parameters (A, B, C): delays = 10; 10; 15; weights = 8.0; 8.0; 6.0;.

A particularly interesting direction of research has been the interaction of metaplasticity with a rarely studied phenomenon called *spike latency* that is an intrinsic property of the integrator type neurons employed as excitatory cells in this study (Izhikevich, 2007). Spike latency is the delay in spike generation that occurs when a neuron is stimulated at near threshold levels. A simple demonstration can be seen in Figures 7A,B. Figure 7A shows a small network of four neurons in which neurons 1, 2, and 3 provide input to neuron 4. In Figure 7B we see the effect of varying levels of stimulation on the firing time of neuron 4 as the connection weights are incremented together in fixed-sized steps. If the input level is barely superthreshold (at 17 mV), neuron 4 spikes at around 30 ms (including connection delays). However, as the input level is increased the firing time of neuron 4 migrates backwards until all three connection weights are saturated.

Spike latency can explain some unusual results in the dynamics of connection weights. If a network such as the one in Figure 7A is repeatedly stimulated with a firing pattern that is congruent with the connection delays then the interaction of STDP with the convergent impulses arriving on neuron 4 produces a strong positive synaptic drive that causes the weights on all three connections to increase to saturation and stay there. However, small changes in the network parameters can produce the effect demonstrated in Figure 7C in which the weight of Connection C first increases and then decreases. This effect was engineered by decreasing the initial weight on C and making the Connection C delay just a little longer than the delays on A and B. In the first 5 s of training the spike arrival time on C occurs *before* spiking of neuron 4, as is also true of Connections A and B. However, as the combined connection weights increase causing the firing time of neuron 4 to migrate backwards, the spike arrival time on C occurs *after* neuron 4 firing, producing synaptic depression on C.

We hypothesize that spike latency is involved in the underlying mechanism that supports the stability of polychronization, and hence in the ability of PNGs to extend. In large networks with recurrent connections, the effect of recurrent input and other factors such as random firing influence the firing probabilities of PNG neurons in response to subsequent activating stimuli, resulting in complex and unpredictable dynamics. Nevertheless, PNGs are able to exist and even extend, despite this input variability that threatens their stability. The neurons in a polychronous group are exposed to a wide range of spatio-temporal input patterns that we refer to as an *input space*. Individual PNG neurons fire in response to only some of these input patterns, and this subset of the input space we term the *active input space*. Input patterns of particular significance in the active input space are those that result from polychronization in neighboring PNG neurons. However, even these polychronising input patterns can occur with considerable jitter in impulse arrival times due to the complex dynamics of the network. It therefore seems to us that a mechanism that expands the size of the active input space (i.e., the range of patterns that produce neural firing) will increase the firing probability of each PNG neuron in response to the current wave of polychronization. Expansion of the active input space should therefore increase the stability of polychronization, resulting in extended polychronization and an increase in PNG size.

**Figure 8:**
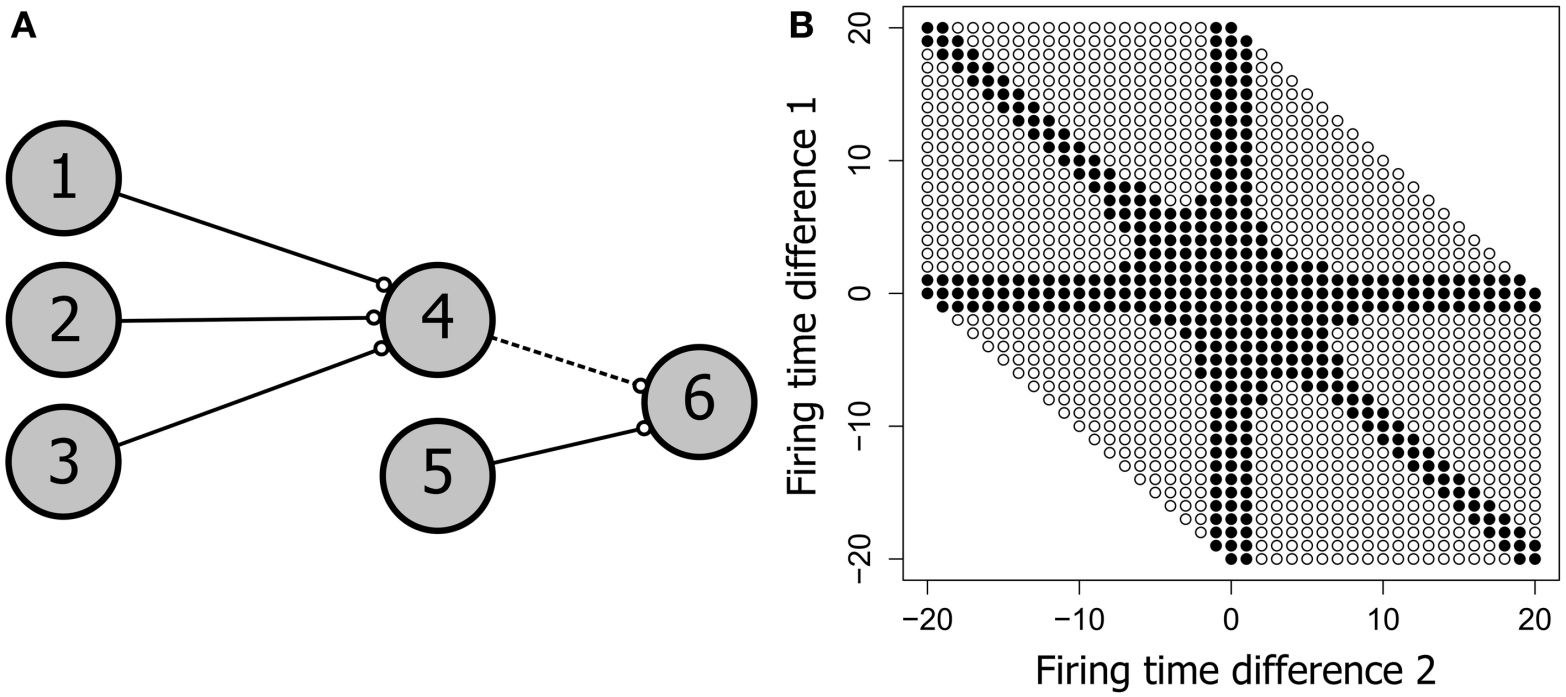
**The neural input space**. **(A)** A potential polychronous group consisting of six neurons. **(B)** The input space of neuron 4, showing the patterns that produced firing of neuron 4 as filled circles and patterns that failed to produce firing as unfilled circles. Neurons 1, 2, 3, and 4 have the same topology as in Figure 7. The two axes represent the firing time differences (*t*1 − *t*2) and (*t*2 − *t*3), where (*t*1, *t*2, *t*3) are the firing times of neurons 1, 2, and 3. All connection weights are initially set to the maximum synaptic weight of 10 mV. Neuron 4 therefore requires at least two convergent inputs to reach firing threshold. The ability of each firing time combination to produce firing of neuron 4 is tested over 400 trials in fixed-sized 250 ms test frames. Consistent firing requires that neuron 4 fire in at least 100 of the 400 test frames. When weights are saturated, just two of the three inputs are required for firing of neuron 4, producing the *arms* in the input space diagram.

Spike latency allows the precise firing timing to be a function of the level of afferent input, potentially allowing an increase in the range of inputs that produce firing. Our current hypothesis is that spike latency allows increased flexibility in the precise timing of neural firing, producing an expansion of the input space for each PNG neuron. To see how this might work, consider the network shown in Figure 8A. This potential polychronous group is composed of six neurons and is derived from the four neuron network of Figure 7. Varying the firing times of the initial three input neurons (1, 2, and 3) produces a wide range of spiking patterns on neuron 4 that together define the input space. With three inputs, this input space is a three dimensional cube that includes the subset of patterns that produce *firing* of neuron 4.

Figure 8B shows the input space of neuron 4. For convenience, the three-dimensional input space has been flattened to two dimensions by taking the difference between each pair of firing times for the three input neurons. Nevertheless, the figure represents the entire input space i.e., all possible spatio-temporal patterns onto neuron 4 that can be generated if each input neuron is allowed to independently vary its firing time in the range 0–20 ms. Each circle in Figure 8B represents a pattern, with filled circles denoting those patterns that produce firing of neuron 4 (the active input space). The larger the proportion of the available input space that is consumed by the active input space, the more flexible the neuron is to jitter in its spatio-temporal inputs.

**Figure 9:**
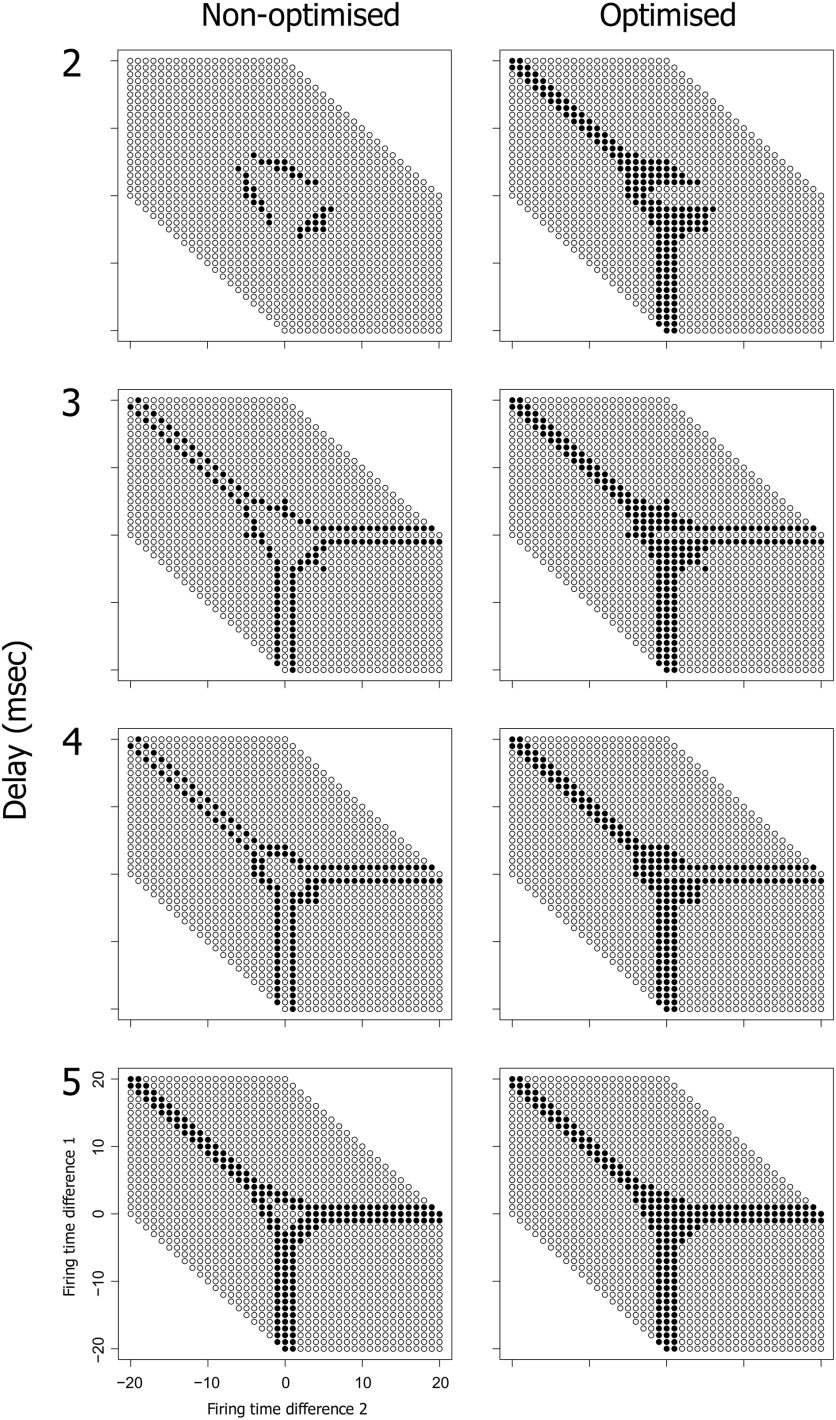
**Optimization of spike latency produces expansion of the input space, potentially allowing a nascent PNG to extend**. The optimization involves systematically varying the weight on connection 1–4 over the range 0–10 mV in order to shift the firing time of neuron 4. Neuron 5 has a fixed 1 ms conduction delay and is fired at a fixed offset of 19 ms relative to the current test frame. The input space of neuron 6 is shown, both with and without optimization and at one of four different delays on the 4–6 connection. There is a phase shift in the input space of neuron 6 as delays increase from 2 to 5 ms (left column). Optimization of neuron 4 spike latency produces an expansion in the input space of neuron 6 (right column).

We can also examine the active input space of neuron 6 relative to these same three input neurons (1, 2, and 3) as shown in Figure 9. The active input space for neuron 6 determines the firing probability of neuron 6 under variable conditions, and hence determines the ability of the potential PNG in Figure 9A to extend beyond neuron 4. The left column of Figure 9 (Non-optimized) shows changes in the input space as the phase is shifted through each of four different delays on the 4–6 connection. Importantly, the active input space for many of these phases can be expanded by shifting the firing time of neuron 4 to a time that is congruent with the 4–6 and 5–6 connection delays [right column of Figure 9 (Optimized)]. These shifts in neuron 4 firing time are produced by changes in the connection weights of the three input neurons (1, 2, and 3) and the effects of spike latency. For now we have performed this optimization for each pattern in the input space by trialing each of ten weight steps on the input neuron connections and selecting weights that produce firing of neuron 6 (if any). An important research question, and one that has yet to be resolved, is whether the interaction of any of the known biologically plausible mechanisms such as metaplasticity and STDP can produce stability enhancement of polychronous groups through a mechanism that optimizes the active input space of each PNG neuron.

## 4. Discussion

The BCM model famously introduced the idea of a sliding modification threshold in which the tipping point for LTP/LTD induction is determined by the average of recent spiking activity in the post-synaptic cell. Many subsequent models of metaplasticity have followed the BCM model in defining a spike-activity-dependent modification threshold, although these models are typically independent of synaptic size and are not able to prevent synapses from becoming arbitrarily large. In the current study we employ a model of metaplasticity in which the modification threshold (θ_*M*_(*t*)) is not spike-activity-dependent but is instead set more directly from the current synaptic weight and the level of a spike-timing-dependent variable, the synaptic drive. An implementation of this model was shown to have a significant effect on the size of polychronous groups in large recurrent networks. An understanding of the underlying mechanism for this enhancement is likely to shed light on the principles of PNG formation and perhaps therefore also on the processes of memory formation and storage.

A spike-timing-dependent learning rule appears to be a significant contributor to synaptic plasticity in many parts of the brain. As shown by (Izhikevich and Desai, 2003), BCM-like behavior can be reproduced with an STDP learning rule using uncorrelated or weakly correlated firing of pre- and post-synaptic cells, provided that the spike interaction model conforms to a variant of nearest-neighbor. Biologically realistic spiking patterns are likely to have both weakly correlated and strongly correlated components, with polychronous firing patterns providing an important example of the latter. With strongly correlated spiking patterns the direction and magnitude of synaptic plasticity is no longer determined by the post-synaptic spike rate alone: spike trains with pre- before post-synaptic spike timings produce an upwards or positive drive on synaptic plasticity, whilst post- before pre-synaptic spike trains with identical firing rates produce the opposite effect, a downwards or negative synaptic drive.

As discussed in Section 2.1.2, our metaplastic mechanism was found to maintain the weight of a single synapse within predefined limits without reaching maximum capped values. However, when translated into a large scale network composed of one hundred thousand synapses this moderating influence was considerably diluted and capping at the global weight limits was no longer achieved. Nevertheless, our modeling results show that metaplasticity has a small but significant effect on the distribution of synaptic weights in the network, producing an overall shift toward larger weights. Networks with metaplasticity show a decrease in the number of pruned synapses, and an increase in the number of saturated and non-saturated synapses (Figure 3). This trend toward stronger weights is particularly noticeable within the PNG connection group where there is a significant preference for saturated weights relative to the non-PNG connection group. However, there is also a significant increase in pruned synapses within this group, in contrast to the overall trend observed in Figure 3.

Other effects of metaplasticity include an increase in the excitatory firing rate, and an increase in the *number* of PNG connections. Much of the increase in the size of the PNG connection group is due to an increase in participating excitatory connections, although both excitatory and inhibitory connections show an increase in participation with metaplasticity (Figure 5). Perhaps the most interesting finding from the current study was the sensitivity of PNG size to these small metaplasticity-induced changes in network parameters. Small changes in weight distributions produced a 16% increase in PNG size, suggesting that factors that alter the network connectivity have a strong influence on the stability of neural circuits based on polychronization. A more refined version of the current metaplastic model with carefully tuned parameters might therefore substantially influence the efficiency of polychronization.

Together these results suggest that neurons that participate in polychronization prefer a smaller number of stronger afferent connections relative to non-participating neurons. A high level account of the effects of metaplasticity on PNG size might therefore be constructed by observing the overall match between the effects of metaplasticity on the synaptic weight distribution (i.e., more saturated and non-saturated weights), and the preference of PNG connections for saturated weights. However, a deeper explanation is required that describes the underlying mechanism whilst accounting for the pruning of PNG connection weights. To this end we have explored a number of avenues such as the effect of metaplasticity on the temporal firing precision, or on the evolution of synaptic weights over time. The effect of spike latency on the active input space of PNG neurons has been a particularly interesting research direction. Initial results show that spike latency allows increased flexibility in the precise timing of neural firing, and that expansion of the active input space for each neuron can be achieved by optimization of spike latency. We speculate that a mechanism that optimizes the active input space of each PNG neuron might produce stability enhancement of polychronous groups through the interaction of metaplasticity with a biologically plausible learning rule such as STDP.

### Conflict of interest statement

The authors declare that the research was conducted in the absence of any commercial or financial relationships that could be construed as a potential conflict of interest.
